# Non-invasive radionuclide imaging of trace metal trafficking in health and disease: “PET metallomics”

**DOI:** 10.1039/d2cb00033d

**Published:** 2022-04-11

**Authors:** George Firth, Julia E. Blower, Joanna J. Bartnicka, Aishwarya Mishra, Aidan M. Michaels, Alex Rigby, Afnan Darwesh, Fahad Al-Salemee, Philip J. Blower

**Affiliations:** School of Biomedical Engineering & Imaging Sciences, King's College London, St. Thomas’ Hospital London UK george.firth@kcl.ac.uk philip.blower@kcl.ac.uk

## Abstract

Several specific metallic elements must be present in the human body to maintain health and function. Maintaining the correct quantity (from trace to bulk) and location at the cell and tissue level is essential. The study of the biological role of metals has become known as metallomics. While quantities of metals in cells and tissues can be readily measured in biopsy and autopsy samples by destructive analytical techniques, their trafficking and its role in health and disease are poorly understood. Molecular imaging with radionuclides – positron emission tomography (PET) and single photon emission computed tomography (SPECT) – is emerging as a means to non-invasively study the acute trafficking of essential metals between organs, non-invasively and in real time, in health and disease. PET scanners are increasingly widely available in hospitals, and methods for producing radionuclides of some of the key essential metals are developing fast. This review summarises recent developments in radionuclide imaging technology that permit such investigations, describes the radiological and physicochemical properties of key radioisotopes of essential trace metals and useful analogues, and introduces current and potential future applications in preclinical and clinical investigations to study the biology of essential trace metals in health and disease.

## Abstract 2

Radioactive isotopes of elements from across the periodic table allow for non-invasive imaging of essential metals in health and disease.

## Graphical abstract

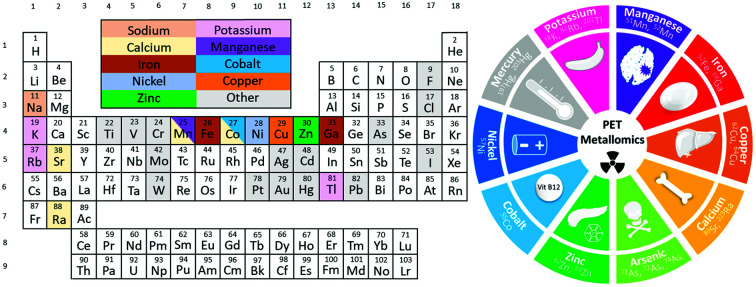

## Introduction

**Fig. 1:**
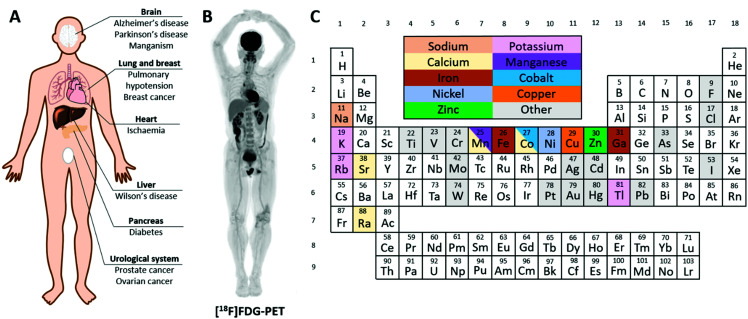
Radioactive metals allow for the non-invasive study of essential trace metals *in vivo* in health and disease. (A) A summary of diseases implicated in metal homeostasis dysregulation that can be studied using radionuclide imaging. (B) Example of a total-body [^18^F]FDG PET scan highlighting glucose avid tissues.^2^ (C) Periodic table colour-coded to highlight elements with useful radioisotopes that can be used to image trace metal biology. Radionuclides with metallomics applications are discussed in individual sections throughout this review.

Trace metals, such as iron, copper, zinc and manganese, are essential for the normal function of living organisms. In health, the absorption, storage, utilisation and excretion of these important metals are under strict control; deficiency or overload adversely affects health. Dysregulation of normal metal homeostasis has been implicated in a number of diseases, including cancer, neurological disorders such as Alzheimer's disease and inflammatory conditions such as rheumatoid arthritis (Fig. 1A), and is receiving heightened interest among biomedical researchers. A diverse range of tools and analytical techniques is available to study the biological behaviour of metals, each with its own strengths and weaknesses. The concentration of trace metals in tissues can be investigated by analytical methods such as inductively coupled-plasma mass spectrometry (ICP-MS), synchrotron based methods such as X-ray fluorescence microscopy, and fluorescent metal-binding probes. These are powerful tools to study metal distribution in tissues and cells and some have been developed into cell-level imaging techniques to determine the cellular, and even sub-cellular, location of trace metals within tissues. For example, mass spectrometric detection methods such as laser ablation (LA) ICP-MS and secondary ion mass spectrometry (SIMS) can provide 2-dimensional maps of trace metal distribution in tissue sections.^1^ However, the majority of these techniques require *ex vivo* tissue samples from autopsy or biopsy and hence are invasive, and the analytical method destructive. They provide a snapshot of concentration and location in time, and each sample is analysed at only one time point. This perspective is necessary to examine the static consequence of chronic and prolonged accumulation of metals in cells and tissues, but not sufficient because it provides no information on the dynamic processes that led to it. In order to provide a perspective on the acute, dynamic handling of trace metals *in vivo*, at the whole body or organ level, non-invasive and non-destructive techniques are needed.

Molecular imaging methods employed clinically in the field of nuclear medicine offer a solution. Nuclear medicine uses picomolar quantities of radioactive substances to study biological processes by three-dimensional, non-invasive dynamic imaging of their distribution in the whole body, exploiting the tissue-penetrating power of gamma radiation. Positron emission tomography (PET) and single photon emission computed tomography (SPECT) are radionuclide imaging techniques that provide three-dimensional information on the spatial and temporal biodistribution of radioactivity in the body. In the clinic, the PET radiotracer fluorodeoxyglucose ([^18^F]FDG) containing the positron-emitting radionuclide ^18^F accounts for over 90% of all scans and remains the standard for the diagnosis of cancer by providing information on glucose metabolism (Fig. 1B). ^99m^Tc, a gamma emitter, is the most commonly used medical radioisotope, accounting for over 90% of SPECT scans and the vast majority of all nuclear medicine scans. A diverse range of ^99m^Tc- and ^18^F-labelled radiopharmaceuticals are employed to study a broad range of biophysical processes, including imaging renal and hepatic function, bone metastases, cardiac perfusion and function, pulmonary ventilation and perfusion. In recent years, emphasis in nuclear medicine has shifted towards development of radiotracers that target specific *molecular* processes using radiolabelled metabolites, receptor-binding peptides or tumour-targeted antibodies. Often the radiolabels used in these biomolecular radiopharmaceuticals are metals (^99m^Tc, ^64^Cu, ^68^Ga, ^89^Zr *etc.*). Well-established examples include ^89^Zr-DFO-trastuzumab and ^68^Ga-PSMA.^3–5^ The principle of using radioactive isotopes of metals for routine diagnostic medicine and for research, in humans and in animal models, is thus well-established. The use of radiometals to study the trafficking and metabolism of the metals themselves, by PET and SPECT imaging, is less well-established because the radiometals most convenient and available to use in nuclear medicine do not match the list of biologically essential metals. Recently however, recognition of the value of using radiometals to image the biology of the metal has grown, and methods to produce useful, imageable radioisotopes of the biologically important metals (*e.g.* manganese, iron, copper and zinc) are being developed. Thus, ‘PET metallomics’ is an emerging paradigm that uses positron- (and gamma-) emitting radiometals to non-invasively study metal trafficking *in vivo*, both in animals and humans.^6^ Though there are other imaging modalities that can provide information on metal trafficking non-invasively, this review is focused specifically on PET and SPECT radiometals. Magnetic resonance imaging (MRI) contrast agents have been discussed where relevant, but are not the main focus of this review.

This review will provide a timely summary of PET and SPECT radiometals that can be implemented to study essential trace metals dynamically and non-invasively across the whole-body, to develop understanding of the links between aberrant metal trafficking and disease, and, potentially, to diagnose disease in patients and monitor the effects of treatment. Established and emerging radionuclides that can image endogenous metals (Fig. 1C), classified by periodic group, will be discussed, including recent developments in radionuclide production and imaging technology that will help pave the way for this emerging field. Questions that can be addressed by such methods, and potential clinical applications, will be discussed.

## Group 1 (Na, K, Rb)

Alkali metals have critical roles in biology. Together, sodium and potassium are responsible for establishing the transmembrane electrochemical gradient in most cells, and facilitate the generation and transmission of action potentials along neurons and into tissues.^7^ Sodium and potassium exist in the ionic +1 oxidation state (Na^+^ and K^+^). Na^+^ is the primary cation of extracellular fluid, with a typical healthy concentration of 135–145 mM.^8^ K^+^ is the most prevalent cytosolic cation in eukaryotic organisms. In humans, 98% of K^+^ is intracellular with an average intracellular concentration of 140–150 mM in healthy cells.^9^ The concentration gradient across the plasma membrane (high intracellular potassium, low extracellular potassium; low intracellular sodium, high extracellular sodium) and the accompanying electrical potential gradient (cytosol negative relative to extracellular space) is driven and maintained mainly by the Na^+^, K^+^-adenosine triphosphatase (Na^+^/K^+^-ATPase). This active, ATP-consuming membrane pump is ubiquitously expressed in the body, but the rate of enzymatic activity and thus the flux of Na^+^ out and K^+^ in, in a ratio of 3 : 2, across the plasma membrane varies in different tissues and organs.^10^ The heart has a greater uptake of K^+^ and other cationic radiotracers compared to other healthy tissues, and this underpins the uptake mechanism of some cardiovascular perfusion imaging agents (see below).

Due to the ubiquitous and essential function of Na^+^ and K^+^, radionuclides of sodium and potassium have been of interest to those investigating the biology of these ions *in vivo*. Several radioisotopes of sodium are available, but few have radiological properties that support imaging. ^22^Na (β^+^, *t*_1/2_ = 2.6 years) has been used to image sodium transport in plants,^11–13^ but has seen limited use in humans due to its long half-life which results in high radiation absorbed dose to subjects receiving it. ^24^Na (β^−^, *t*_1/2_ = 15 hours) has a shorter half-life that is more suited to studying sodium trafficking, but its poorly-penetrating beta emission limits its use to *in vitro* measurements with a beta detector. Many of the radioisotopes of potassium are likewise unsuitable for modern imaging techniques given their β^−^ decay. The short half-life of ^38^K (*t*_1/2_ = 7.6 min) and its requirement for an on-site cyclotron producing a high-energy (*ca.* 30 MeV) proton beam prevent widespread availability. It was, however, used for imaging studies in pre-clinical models at the dawn of PET imaging^15,16^ and in-human investigations were performed to evaluate its utility as a perfusion imaging agent, exploiting its high and rapid accumulation in myocytes *via* the Na^+^/K^+^-ATPase.^15,17^ The conclusions drawn from these studies were that ^38^K accumulates readily in cardiac tissue and has lower uptake in the liver compared to other myocardial perfusion imaging agents such as [^62^Cu]Cu–PTSM (an established perfusion imaging agent at the time of investigation).^17^ Advances in technology supporting PET imaging may warrant revisiting this radionuclide for its classical use of cardiovascular imaging or for studies of changes to the *in vivo* biodistribution of K^+^ in different disease states. However, its short half-life and cyclotron production will hinder widespread adoption.

**Fig. 2:**
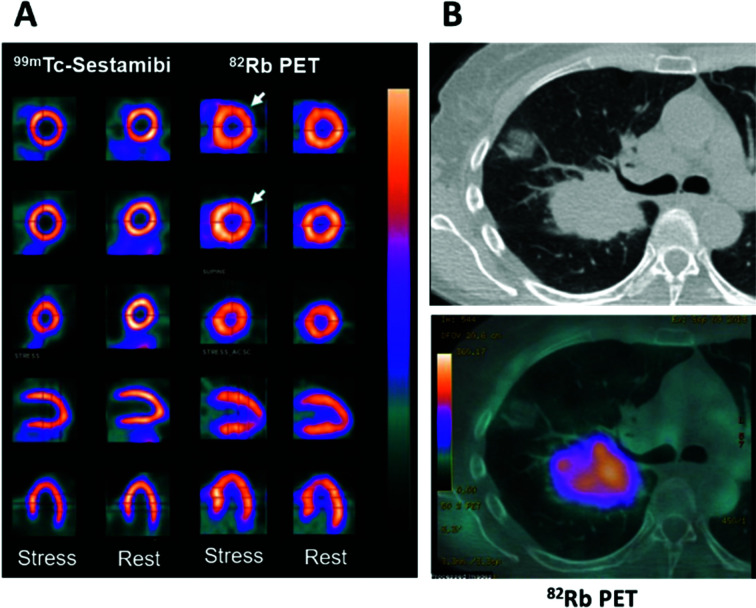
Applications of potassium mimetics in nuclear medicine. (A) Representative myocardial perfusion imaging with ^99m^Tc-Sestamibi SPECT and ^82^Rb-PET.^14^ From top to bottom: axial apical, mid and basal sections, coronal and sagittal slices. (B) Clinical CT scan of a lung cancer patient (top) with a corresponding axial section from a ^82^Rb PET scan (bottom) (courtesy of A. Groves, UCL). Myocardial perfusion images are adapted and reproduced with permission under a Creative Commons Attribution (CC-BY) License from ref. 14, *Front. Med*., copyright© 2015.

The poor availability of sodium and potassium imaging radionuclides can be partially mitigated by use of surrogate radioisotopes of chemically analogous metals. ^82^Rb and ^201^Tl fulfil this role for potassium. ^82^Rb (β^+^, *t*_1/2_ = 76 s) is the only group 1 radionuclide that has seen significant use in nuclear medicine. The key to its widespread use is its production *via* the ^82^Sr/^82^Rb generator, which has a shelf-life of ∼1 month. ^82^Rb formed by decay of ^82^Sr can be eluted as [^82^Rb]RbCl with physiological saline directly into a patient *via* intravenous (i.v.) infusion – a necessity as the rapid decay does not permit manipulations with syringes or radiochemistry. The short half-life of ^82^Rb allows repeated dosing and imaging studies to be performed, for example, preceding and following a therapeutic intervention, while keeping the radiation dose to the patient low. Consistent with its periodic location immediately below potassium, and despite a slightly larger ionic radius, Rb^+^ is able to enter cells *via* the Na^+^/K^+^-ATPase, one of many transporters responsible for potassium influx, and therefore has potential applications in the field of metallomics as a K^+^ mimic, as well as myocardial perfusion imaging. The *in vivo* pharmacokinetics and pharmacodynamics of ^82^Rb have made it a routine option for imaging myocardial perfusion, with initial ^82^Rb uptake in metabolically active tissue proportional to blood flow. This results in comparable quality images to the traditional radiotracers used for SPECT imaging (Fig. 2A).^14,18^

The utilisation of ^82^Rb has recently expanded beyond myocardial perfusion imaging into the field of clinical oncology where it has been used to image tumour perfusion (Fig. 2B). Critically, the inferences drawn from these investigations require further probing to distinguish tumour cell uptake of ^82^Rb compared to accumulation in the tumoural interstitial fluid. Notably, the first in-human investigations have indicated that prognostic information can be gathered from ^82^Rb imaging of tumours. Jochumsen *et al.* demonstrated increased uptake in higher grade prostate cancer compared to lower grade.^19^ Like all blood–borne radiotracers, the tissue accumulation is governed both by perfusion and the abundance of the specific molecular target or binding mechanism. Which of these contributors dominates, and hence whether or not ^82^Rb imaging can quantify Na^+^/K^+^-ATPase activity (either in cardiac or tumour imaging), remains to be fully determined. In the aforementioned prostate cancer study, ^82^Rb uptake did not correlate with cell or Na^+^/K^+^-ATPase optical density; on the other hand other studies suggest that ^82^Rb is not a pure perfusion tracer either.^20^ These findings warrant further investigation, particularly whether Na^+^/K^+^-ATPase activity is the driving mechanism for uptake and whether tissue uptake reflects Na^+^/K^+^-ATPase activity or merely perfusion. Repurposing ^82^Sr/^82^Rb generators for oncological imaging may be a future trend in clinical oncology offering a complementary imaging method to ^18^F-FDG. The short half-life and rapid clearance could enable greater prognostic information with minimal increase to the scan time and radiation absorbed dose to patients, helping to stratify patients more effectively.

The short half-life of ^82^Rb is advantageous as it permits multiplexed imaging (*i.e.* imaging several radiotracer distributions rather than just one, which may become possible using Total Body PET^2^) with other radioactive metals to map their trafficking in health and disease. However, it also restricts imaging to very early time points, where perfusion may be the main determinant of uptake, rather than the molecular trapping mechanism and thus negating any molecular information value. SPECT imaging with the longer half-life ^201^Tl may offer a solution to this problem. Although not endogenous to the human body, and toxic in large quantities, thallium in the ionic +1 oxidation state (Tl^+^) can be used as a surrogate marker of K^+^. Due to the similarity of its ionic radius (150 pm) to that of K^+^ (138 pm), it has similar affinity for potassium channels such as the Na^+^/K^+^-ATPase. Thus, cyclotron-produced ^201^Tl (γ, *t*_1/2_ = 3.04 days, 69–81 keV X-rays), intravenously administered in nano- to picomolar quantities as thallous chloride ([^201^Tl]TlCl), has historically been used in the clinic to image myocardial perfusion with gamma scintigraphy and SPECT.^21^^201^Tl is rapidly cleared from the blood and localises primarily to the heart and skeletal muscle with clearance through the kidneys.^22^ Poor delivery from impaired blood flow (ischaemia) or damaged tissue gives rise to ‘cold spots’, and thus provides clinically relevant information that is used in the visualisation and diagnosis of myocardial infarction. ^201^Tl imaging can in principle provide information on Na^+^/K^+^-ATPase activity but this remains to be validated *in vivo* and clinically.

The utility of ^201^Tl is not limited to just myocardial imaging; clinical investigations of glioblastomas,^23^ lymphoma, breast and lung cancers have also been performed.^24^ These studies, largely from the 1980's and 1990's, demonstrated use in distinguishing benign from malignant lesions and evaluating chemotherapy response and tumour malignancy. Additional studies in the context of imaging potassium biology in cancer are required, especially given that the Na^+^/K^+^ ATPase could be a therapeutic target for cancer treatment.

Although ^201^Tl has played an important historical role in the development of nuclear medicine, it is much less widely used now than in the past, and has largely been replaced as a myocardial perfusion imaging agent by ^99m^Tc-labelled tracers, which accumulate in myocardium by mechanisms unrelated to Na^+^/K^+^ ATPase activity.^18^ The disadvantages of ^201^Tl are largely associated with its emission properties. The low energy of the photon emissions causes high soft tissue attenuation and thus poor image quality. In addition, the long half-life, while offering the advantage of international transport and late time-point imaging, also imparts high radiation dose to subjects; likewise the unusually high number (*ca.* 37 per decay) of Auger electrons emitted as part of the decay process add substantially to the radiation dose absorbed by the cell in which the radionuclide resides, inducing significant cellular toxicity.^25,26^ Nevertheless, some institutes still rely on ^201^Tl for imaging myocardial viability for its high specificity and sensitivity for coronary artery disease,^27^ and there is renewed interest in the radiobiology of ^201^Tl because the aforementioned Auger electron emissions may offer radionuclide therapy applications.^28,29^

## Group 2 (Mg, Ca, Sr, Ra)

Like group 1, group 2 lacks radioisotopes of essential metals that are suitable for imaging. The majority find applications as surrogates for imaging calcium. The beta emitter ^89^Sr (β^−^, 100%, *t*_1/2_ = 53 days), administered as strontium chloride, has been used for many years as a palliative therapy for painful bone metastases in cancer. Accumulation of strontium in sites of active bone mineral deposition (*e.g.* bone metastases) is thought to be driven by calcium mimicry. However, ^89^Sr lacks positron and gamma emissions and so cannot be imaged. The gamma emissions of ^85^Sr (γ, 100%, *t*_1/2_ = 65 days) can in principle be used to determine its quantitative biodistribution but the long half-life limits clinical applications. ^223^Ra (α, 94%, *t*_1/2_ = 11.4 days), used similarly in the form of radium chloride, also accumulates in sites of bone mineralisation and is a potent therapeutic in bone metastases.^30^ Although it has some imageable gammas (<2% abundance), a substantial radiation dose from its alpha emissions make it an unrealistic tool for imaging calcium flux. Manganese and cobalt have also been reported to display calcium mimetic behaviour,^31–33^ but given that these metals are endogenous they have their own regulatory pathways which in some cases may be shared with other metals, making their use for the study of calcium trafficking unrealistic (both of these metals, and the evidence for their similar behaviour to calcium, are discussed in respective sections below).

Positron emitting radioisotopes of magnesium have half-lives <10 seconds and so cannot be practically imaged.

## Group 3–5 (Ti, V, Sc)

Groups 3 through 5, although containing key radionuclides used within nuclear medicine such as ^89^Zr (long half-life positron emitter used as an antibody^3,34^ and cell^35^ radiolabel) and ^90^Y (high energy beta emitter used in radionuclide therapy), almost entirely lack biologically relevant metals. Vanadium is essential for some organisms,^36^ but is not currently recognised as an essential metal in humans. Interest in vanadium lies in its application in vanadium-based drugs, such as complexes of the vanadate (H_2_VO_4_^−^) anion or the vanadyl (VO^2+^) cation. For example, orally administered vanadium complexes have shown efficacy in the treatment of hyperglycaemia in diabetes due to their insulinomimetic behaviour,^37^ but mechanisms remain poorly characterised. Gastrointestinal absorption rate and tissue distribution of these complexes could be addressed with radiovanadium PET. Positron emitting radioisotopes of vanadium – ^47^V (β^+^, *t*_1/2_ = 33 min) and ^48^V (β^+^, 50%, *t*_1/2_ = 16 days) – are available and could be used for these purposes, but have seen little use so far.

The generator-produced ^44^Sc (β^+^, 94%, *t*_1/2_ = 3.97 h) and its therapeutic counterpart ^47^Sc (β^−^, 100%, *t*_1/2_ = 3.35 days) have attracted interest in nuclear medicine as a theranostic pair (an imaging radionuclide and a therapeutic radioisotope of the same element),^38^ though in the context of this review scandium lacks biological significance. Titanium also has a useful radioisotope for PET imaging – ^45^Ti (β^+^, 85%, *t*_1/2_ = 3.08 h),^38^ but has little application in PET metallomics except perhaps in relation to imaging titanium toxicity from titanium implants.^39^

## Group 6 (Cr, Mo, W)

Chromium in its trivalent oxidation state is found in most foods and nutrient supplements. The highest levels in human tissues are found in the kidney and liver.^40^ Whether chromium is an essential element in humans remains a topic of debate.^41^ Several studies have shown that chromium supplementation has a positive impact on glycaemic control in diabetes, though conflicting data have also been reported.^42^ Hexavalent chromium is toxic and was shown to be a human carcinogen when inhaled.^43^ Homeostatic mechanisms that control chromium *in vivo* are unknown, but low-molecular-weight chromium-binding substance (LMWCr, also known as chromodulin) and transferrin are likely to play a role. ^49^Cr (β^+^, 93%, *t*_1/2_ = 42.3 min) has attractive emission properties for PET imaging of chromium biology, at least in animal models, though its radioactive daughter ^49^V (*t*_1/2_ = 329 days) limits its use. The gamma rays produced by ^51^Cr (γ, 9.9%, *t*_1/2_ = 27.8 days) are of too high energy, and its half-life is too long, for imaging applications. However, it has seen historical use in nuclear medicine for non-imaging tracer applications in haematology and nephrology.^44^

Molybdenum is found in the body in approximately ten-fold greater quantity compared to chromium (9.3 *vs.* 1.8 mg respectively in a 70 kg human) and similar quantity to manganese (12 mg).^41^ Molybdenum is essential for humans; sulfite oxidase and xanthine oxidase both require it for their function.^45^ Notable molybdenum radioisotopes include ^90^Mo (β^+^, 24.9%, *t*_1/2_ = 5.6 h), which is the parent isotope for niobium-90 (β^+^, 53%, *t*_1/2_ = 14.6 h), and ^99^Mo (β^−^, *t*_1/2_ = 66 h), widely known in nuclear medicine as the parent in the ^99m^Tc generator. Neither of these molybdenum isotopes are well-suited for metallomics-based imaging applications.

To date, no essential role for tungsten in humans has been recognised, though some microbial enzymes that require it have been identified.^45^ Chronic environmental overexposure to tungsten, particularly of those working in metallurgy, may induce toxicity. Beta emitters such as ^185^W (β^−^, *t*_1/2_ = 75.1 days) and ^187^W (β^−^, *t*_1/2_ = 23.7 h) can be used for *ex vivo* radioactivity measurements of tungsten trafficking.^46^^187^W has low energy gamma emissions (72–134 keV)^47^ that may have SPECT potential.

## Group 7 (Mn)

Manganese, predominantly found in its +2 oxidation state in biological environments, is an essential metal with key roles such as defence against reactive oxygen species (ROS), not only in enzymes such as manganese superoxide dismutase and catalase but also in non-enzymatic forms.^48–51^ It also has crucial roles in reproduction,^52^ bone growth,^53^ the urea cycle,^54^ immune function^55^ and blood glucose control.^56^ The proteins that work in unison to mediate manganese import, export and storage are poorly understood. Without a reliable fluorescent probe or medical imaging agent tailored to manganese, our understanding of manganese handling has not progressed as fast as that of copper and zinc.

Disruption of manganese homeostasis can lead to overload and toxicity, *i.e.* manganism, a parkinsonian-like movement disorder characterised by chronic overload of manganese in the basal ganglia of the brain.^57–59^ Mutations in manganese transporters such as SLC30A10 (ZnT10) and SLC39A14 (ZIP14) disrupt the normal regulation of manganese absorption in the gut and excretion from the liver respectively. This leads to an increased concentration of manganese in the blood and subsequent deposition of manganese in the brain.^60^ Elevated concentrations of Mn(ii) are detectable by MRI because of its paramagnetism, forming the basis for the clinical gold standard technique for diagnosing manganism. Both Mn(ii) and Fe(iii) cause hypointensity on *T*_2_-weighted MRI scans; however, *T*_1_-weighted imaging allows differentiation between Mn(ii) and Fe(iii) as only Mn(ii) leads to hyperintensity. Interestingly, similar MRI brain findings have been reported in smelting workers, who showed the same MRI signature characteristic of manganese overexposure despite lacking clinical symptoms of manganese intoxication.^61–65^ Other causes of manganism that present with similar MRI brain appearances include overexposure of workers in certain occupational/environmental contexts, such as mining and welding,^66^ prolonged periods of parenteral nutrition,^67^ chronic liver disease involving impaired hepatobiliary excretion of manganese,^68^ and, among drug addicts, intake of manganese-contaminated ephedrone.^69,70^

The late 90s saw the introduction of MRI with administered exogenous free Mn^2+^, later termed manganese-enhanced MRI (MEMRI), as a tool for peripheral neuron tracing,^71^ based on the characteristic ability of Mn^2+^ ions to move in an anterograde manner along neurons. The mechanism controlling this transport remains unknown, though evidence from mice deficient in L-type Ca^2+^ channel 1.2 (Ca_v_1.2) suggests Ca mimicking may be involved.^72^ The use of MEMRI in neuroscience has been reviewed.^73^ The large quantities of manganese that must be administered for adequate MR contrast, which are greatly in excess of those found biologically, unavoidably perturb homeostasis of manganese and probably other trace metals, and may be toxic. MEMRI is unlikely, therefore, to provide a clinically useful or indeed realistic summary of normal manganese handling *in vivo*. Manganese-based complexes are of interest as a replacement to Gd-based contrast agents that have come under intense scrutiny over the years due to the prevalent off-target accumulation of toxic exogenous Gd^3+^. However, the design and application of manganese-based MRI contrast agents is beyond the scope of this review and has been covered by others.

**Fig. 3:**
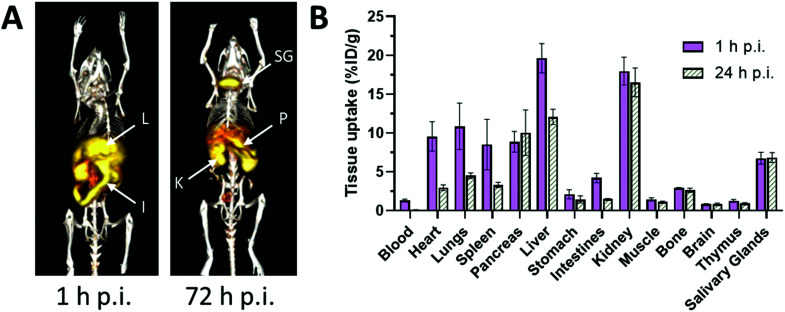
Biodistribution and pharmacokinetics of ^52^Mn, administered i.v. as MnCl_2_, in healthy mice. (A) Representative serial maximum intensity projection (MIP) PET images of healthy mice at 1 and 72 hours p.i. ^52^Mn is primarily distributed to abdominal organs such as liver (L), kidney (K) and intestines. At 72 h p.i., ^52^Mn is found predominantly in the kidneys, pancreas (P) and salivary glands (SG). (B) *Ex vivo* biodistribution of ^52^Mn in aqueous solution following i.v. injection at 1 and 24 h p.i. Adapted with permission under a Creative Commons Attribution (CC-BY) License from ref. 79, *PLoS One*, copyright© 2017.

**Fig. 4:**
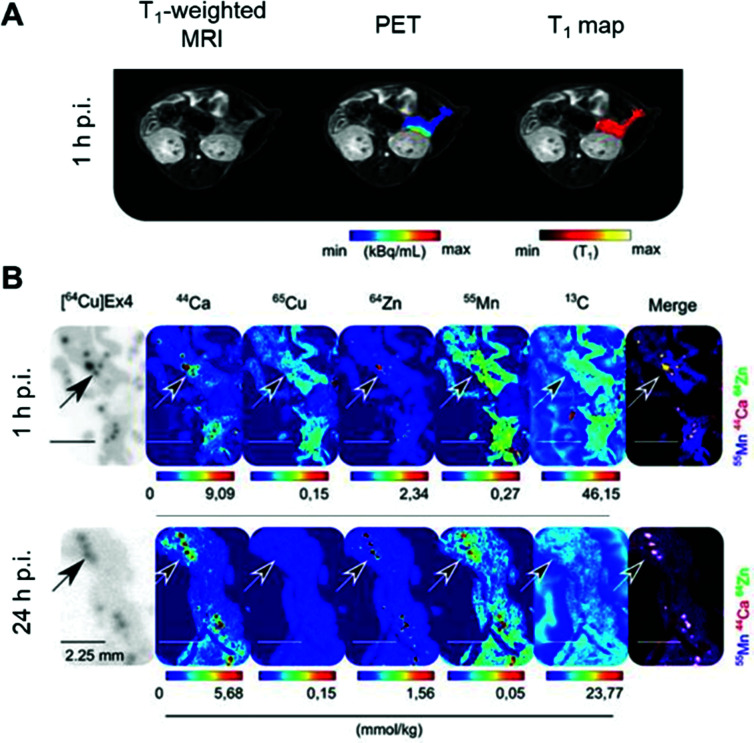
Assessment of β-cell mass and function by PET/MRI. (A) PET/MRI images of the pancreas in mice following co-injection of the β-cell specific radiotracer [^64^Cu]Cu-NODAGA-^40^Lys-Exendin-4 and paramagnetic MnCl_2_. (B) LA-ICP-MS images revealed typical metal tissue distribution for the pancreas, notably high Zn concentrated in the islets (highlighted by arrows) co-localised with [^64^Cu]Ex4 autoradiographs. Interestingly, Mn was found predominately in the exocrine tissue at 1 h p.i., and transitioned to islets by 24 h p.i. Adapted with permission under a Creative Commons Attribution (CC-BY) License from ref. 82, *Theranostics*, copyright© 2020.

Radionuclide imaging offers an alternative and complementary approach to MRI to investigate *in vivo* acute dynamic trafficking of manganese. Several positron emitting radioisotopes of manganese, with emissions and half-lives suitable for applications in PET imaging, have become available in recent years: ^52m^Mn (21.1 min), ^51^Mn (46.2 min), and ^52g^Mn (also referred to as ^52^Mn, 5.6 days). The long-lived ^52^Mn is suited to imaging biological processes with a relatively long half-life, including the labelling of cells,^74^ antibodies^75^ and nanoparticles,^74^ and has been hailed as an emerging option for immunoPET with potential advantage over the currently widely-used ^89^Zr, which has a slightly shorter half-life (3.3 d). The production of ^52^Mn has recently been optimised by Fonslet *et al.*, and can now be performed at facilities with access to a modest energy medical cyclotron (16 MeV) *via* the ^52^Cr(p,n)^52^Mn reaction.^76^ Although several centres worldwide now produce ^52^Mn for research purposes, and owing to its long half-life, it can be shipped internationally, few biological studies of ^52^Mn PET have been published. They include studies that reported the biodistribution of ^52^Mn administered intravenously as manganese(ii) chloride in mice, rats and non-human primates.^33,77–80^ Uptake was highest in pancreas, salivary glands and, at early time points, myocardium (Fig. 3). The significant uptake of manganese in the pancreas, seen in both PET and MRI studies, may be of particular interest for the non-invasive assessment of β-cell mass. Pharmacological modulation of ^52^Mn uptake was achieved in isolated islets and the global pancreas *in vivo* with voltage-dependant calcium channel (VDCC) inhibitors and stimulators.^33^ These studies, combined with reduced ^52^Mn uptake in streptozotocin (STZ)-induced diabetic mouse pancreata, provide evidence that manganese import is mediated by VDCCs and can be used to estimate β-cell mass. STZ treatment has been shown to impact pancreatic vasculature raising the possibility that decreased perfusion, rather than changes in β-cell mass and function, may underlie reduction in manganese uptake.^81^ What is perhaps overlooked is the assumption that radiomanganese and paramagnetic manganese localise primarily to the endocrine component of the pancreas compared to the exocrine – a necessity for determining β-cell mass and function. A recent PET/MRI approach to assess β-cell mass and function was carried out using a radiolabelled GLP-1R antagonist, [^64^Cu]Cu-NODAGA-^40^Lys-Exendin-4, and paramagnetic manganese as MnCl_2_, which was then correlated with autoradiography and LA-ICP-MS (Fig. 4).^82^ LA-ICP-MS images revealed that initial manganese accumulation is inversely related to the β-cell specific radiotracer at 1 h p.i. However, manganese translocates from the exocrine pancreas to the endocrine pancreas by 24 h p.i. This has implications for MEMRI protocols for β-cell mass assessment showing that only late time points actually reflect the secretory function of the pancreas. As an example of possible use of radionuclides in addressing these issues, it would be possible to carry out a similar study correlating acute dynamics of manganese with ^52^Mn-PET and autoradiography, using picomolar quantities of contrast agent (2000 times lower concentration than MEMRI) that are less likely to perturb manganese handling, alongside chronic manganese concentration measurement by LA-ICP-MS, in healthy and diabetic pancreata. This could then be extended to longitudinal assessment of transplanted β-cells survival and function, as islet transplantation therapy results in temporary insulin independence for patients with type 1 diabetes.

The limitations of MEMRI applied to neural tracing, discussed above, could in principle be overcome by use of ^52^Mn PET, although the low spatial resolution of PET imaging would severely limit its use in small animals; this would be a far less significant limitation in humans. Recent studies with ^52^Mn-PET have indeed mapped the movement of manganese along the mesolimbic and nigrostriatal pathways of the brain,^83^ as well as olfactory uptake into the frontal cortex and amygdala following intranasal administration in non-human primates.^80^ Neuronal tract tracing with PET with ^52^Mn in the clinic might have widespread applications, from imaging neuronal repair following surgery to neuronal development and impairment. The shorter-lived (and hence preferable for dosimetry reasons) ^51^Mn could also be used as it has fewer unwanted gamma emissions but its use may be limited by its long-lived gamma-emitting daughter ^51^Cr.

Applications of manganese-PET in cardiology may be of interest following the observation that high myocardial uptake is observed up to 24 h p.i. This has enabled imaging of myocardial infarcts in dogs.^84^

## Group 8 (Fe)

Iron is an important endogenous metal that mediates critical biological processes including oxygen delivery *via* the iron-containing porphyrin haem in haemoglobin, oxidation *via* cytochrome P450 and oxidative phosphorylation *via* FeS clusters in the electron transport chain. A human adult contains ∼3–5 g of iron, 80% of which is found in erythrocyte haemoglobin.^85^ Free, labile/aquated iron is harmful for the body due to its involvement in the generation of ROS species through Fenton reactions. Therefore, iron, like many other endogenous metals, is highly regulated in the body and sequestered as metalloprotein complexes.^86,87^ Iron transitions between redox states of +2 and +3 in the body allowing its transport *via* transferrin to demanding tissues and cells. Non-haem iron is transported into cells by two mechanisms: transferrin (Tf)-bound iron uptake, *via* receptor-mediated endocytosis after binding to transferrin receptor 1 (TfR1), and non-Tf-bound iron (NTBI) uptake. Under physiological conditions, almost all circulating iron in plasma is bound to Tf,^88^ the iron-binding sites of which are normally about 30% saturated. Below 16% saturation indicates iron deficiency, and >60% is a sign of iron overload. The trafficking of iron has been studied to understand its homeostatic regulation under normal conditions and how this goes awry in associated diseases.^89^ For example, the varying concentration of iron in the brain is a subject of investigation of its impact on neurodegenerative disorders such as Wilson's, Alzheimer's and Parkinson's diseases.^90,91^ However, very few imaging studies have been performed due to lack of ideal iron radioisotopes. To investigate the whole body dynamics of iron handling, three radioisotopes of iron have potential use but all are far from ideal. ^55^Fe (γ, *t*_1/2_ = 2.73 years) and ^59^Fe (γ, *t*_1/2_ = 44 days) are gamma emitters with long half-lives that are non-ideal because of high radiation doses. The cyclotron-produced positron emitting ^52^Fe (β^+^, 55%, *t*_1/2_ = 8.28 h) has an optimal half-life and positron branching ratio for PET imaging, but interpretation of results remains complicated due to its decay to the positron emitting ^52m^Mn (β^+^, 96%, *t*_1/2_ = 21 min). PET is unable to differentiate between the two radionuclides, and images represent a summed biodistribution of ^52^Fe and ^52m^Mn.^92^ Tissue-dependent correction factors for humans have been described by Lubberink *et al.*,^92^ although its complex decay continues to make measurements extremely challenging. Nevertheless, it has seen applications in imaging cerebral uptake in Wilson's disease,^93^ studying ^52^Fe uptake in blood and bone marrow related disorders, tracking the life cycle of red blood cells^94–96^ and determining the pharmacokinetics of iron supplements.^92,97^

In a bid to find a radioactive isotope that gives a useful readout of iron biodistribution without unfavourable decay modes, surrogate markers, notably radioisotopes of gallium, have been studied. Although gallium shares only some of the biological characteristics of iron, it has some highly suitable radioisotopes with a range of nuclear properties that make them attractive for studying limited aspects of iron metabolism provided the differences between iron and gallium are understood and taken into account. Ga^3+^ shares a similar ionic radius with Fe^3+^ (62 pm *vs.* 64.5 pm respectively) in hexacoordinated complexes,^98^ and hard ligands containing oxygen and nitrogen donor atoms are favoured by both ions. The similarity between Fe^3+^ and Ga^3+^ was used as inspiration in the development of tris(hydroxypyridinone) (THP) chelators for gallium; they are now utilised in the clinic as ^68^Ga-THP-PSMA to detect prostate cancer.^5,99–101^

^66^Ga (β^+^, 57%, *t*_1/2_ = 9.5 h), ^67^Ga (γ, *t*_1/2_ = 78 h), ^68^Ga (β^+^, 89%, *t*_1/2_ = 68 min) and ^72^Ga (β^−^, γ, *t*_1/2_ = 14.3 h) are the most common radioisotopes of gallium with half-lives suitable for performing nuclear imaging studies. ^67^Ga is a gamma photon and Auger electron emitter, making it a useful radionuclide in SPECT imaging and potentially as a radionuclide therapy.^102,103^ It is produced by high-energy proton irradiation of an enriched ^67^Zn target using a cyclotron. Its long half-life is compatible with imaging studies over several days which is useful for the detection of lymphoma,^104,105^ inflammation^106^ and infection.^107^ However, PET is often favoured over SPECT due to its superior resolution and easier dynamic acquisition in preclinical settings. The advantage of economical and steady supply of ^68^Ga, due to its generator-based production from ^68^Ge (*t*_1/2_ = 271 d), has led to its ubiquity in imaging centres without access to a medical cyclotron. ^68^Ga decays to ^68^Zn *via* positron emission (89%, 1.89 MeV, *t*_1/2_ = 68 min) and, although its half-life is much shorter than ^67^Ga, it is sufficient for *in vivo* investigations up to 4 hours.

Like Fe^3+^, Ga^3+^ is rapidly sequestered in the blood by serum proteins, mainly transferrin. Ga also binds to lactoferrin, a protein released by neutrophils at sites of inflammation and which has higher iron affinity than transferrin.^108^ Coupled with the high expression of TfR1, trafficking *via* transferrin is believed to be the basis for the use of [^67^Ga]Ga–citrate for imaging infection and inflammation, particularly for identifying infection sites in fever of unknown origin (FUO) patients. In the 1980s, the ^67/68^Ga transferrin and lactoferrin complexes were used extensively to investigate tumours and their uptake mechanism.^109,110^ Lactoferrin-bound Ga has been also used to track lymphocytes at the site of inflammation.^106^^67^Ga-citrate has shown increased uptake at sites of oedema.^111^

Uptake of ^67/68^Ga in infection sites may also be due to binding to siderophores – small iron-chelating molecules secreted by bacteria and fungi to enable them to scavenge iron. Many siderophores form gallium complexes that are isostructural with the iron complexes. For example, both the Fe^3+^ and Ga^3+^ complexes of the siderophore desferrioxamine B (DFO) are taken up by bacteria, offering potential for imaging infection.^112–116^

The above examples illustrate ways in which gallium radionuclides can serve as iron surrogates. However, there are of course profound differences between gallium and iron that need to be accounted for. Unlike Fe^3+^, Ga^3+^ cannot be reduced to the +2 oxidation state in physiological conditions. Therefore, Ga^3+^ does not compete with Fe^2+^ to bind to haemoglobin and myoglobin,^117^ nor is it transported by NTBI mechanisms (*e.g.* DMT-1 and ZIP14). Pharmacokinetic differences have been demonstrated between [^67^Ga]Ga-citrate and [^59^Fe]Fe-citrate; uptake of the two tracers in bone, spleen and liver is similar, but there are significant differences in tumour (lymphoma and myeloma) and kidney uptake.^118^

The vast majority of imaging studies of radiogallium trafficking have been performed with [^67^Ga]Ga-citrate. Other forms of administered radiogallium, including unchelated forms (acetate, chloride) may result in differing kinetics of transchelation to transferrin, which may complicate interpretation of scans. In the absence of effective chelators, the biodistribution tends to vary, with higher uptake in liver and spleen sometimes seen, possibly due to gallium hydroxide colloid formation which is hard to control reproducibly in the absence of chelators.^113,119,120^

## Group 9 (Co)

The human body contains approximately 1 mg of cobalt, 85% of which is in the form of vitamin B12. Cobalamin (vitamin B12) is characterised by a porphyrin-like corrin nucleus consisting of four pyrrole subunits and a 5,6-dimethylbenzimidazole pendant arm, which together chelate the Co(iii) ion in an octahedral geometry. Cobalamin mediates crucial biological processes including DNA synthesis and fatty acid and amino acid metabolism. Therefore, there is significant value in studying the pharmacokinetics and pharmacodynamics of this important vitamin in health and in cancer and other pathologies.^121^ The Schilling test,^122^ a historic test for cobalamin deficiency whereby the patient is given an oral dose of cobalamin labelled with a long-lived cobalt radioisotope, ^57^Co or ^58^Co (half-life 272 days and 71 days respectively, hence unsuitable for imaging), followed by urine sampling (no imaging was involved). Imaging with a suitable radionuclide of cobalt would produce additional insight into cobalt and B12 metabolism, but this potential has not been exploited to date.

There are several radioactive isotopes of cobalt, including the aforementioned ^57^Co and ^58^Co as well as an interesting therapeutic option, the Auger electron emitter ^58m^Co (*t*_1/2_, 9.04 h). Only ^55^Co (β^+^, 76%, *t*_1/2_ = 17.5 h) is suitable for radionuclide imaging. It is produced using a deuteron beam (8.5 MeV) from a cyclotron *via* the ^54^Fe(d,n)^55^Co reaction.^123–125^ A limitation on the widespread adoption of this production method is the low natural abundance, and hence high cost, of the target ^54^Fe. Other production methods are available using, for example, nickel targets, but these are often associated with the production of a larger number of radioactive impurities (*e.g.* the long lived ^56^Co (77 d) and ^57^Co (271.8 d)). The half-life of ^55^Co is amenable to the radiolabelling of structurally diverse targeting molecules including peptides and nanobodies, mostly using macrocyclic bifunctional chelators based on DOTA,^123,126,127^ NOTA,^123^ and linear complexes such as EDTA.^128^

**Fig. 5:**
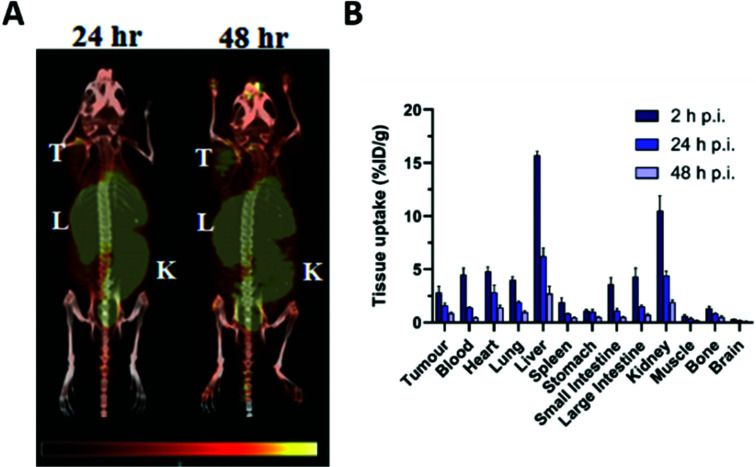
Imaging cobalt trafficking with ^55^Co-PET in a subcutaneous colon cancer mouse model. Biodistribution of ^55^Co in female Nu/Nu mice bearing subcutaneous HCT-116 colon tumours on the upper right flank. (A) PET images at 24 h and 48 h post i.v. administration of ^55^CoCl_2_ demonstrate uptake in the tumour (T) and clearance through the liver (L), kidney (K), and intestines. (B) *Ex vivo* biodistribution at 2, 24 and 48 h p.i. Adapted with permission under a Creative Commons Attribution (CC-BY) License from ref. 111, *Mol. Imaging*, copyright© 2015.

Application of ^55^Co as an imaging tool to study cobalt trafficking has scarcely been implemented. Mastren *et al.* investigated the biodistribution of ^55^CoCl_2_ in mice bearing subcutaneous HCT-116 colon cancer tumours as a control for their ^55^Co-labelled radiopharmaceuticals.^123^ PET imaging at 24 h and 48 h post i.v. administration highlighted liver, kidney and intestinal uptake (Fig. 5A). Uptake was also observed in the heart and tumour. *Ex vivo* biodistribution revealed that ^55^Co retention gradually declined in all tissues from 2 h to 24 h and 48 h p.i. (Fig. 5B). The PET images also highlighted the poor imaging resolution associated with this radionuclide due to its high positron energies (1021 keV, 26% and 1499 keV, 46%). Heart uptake of ^55^Co has been tentatively attributed to analogy in its transport to calcium. Despite low blood–brain barrier (BBB) penetration in healthy subjects, PET imaging of the brain in multiple sclerosis (MS),^129^ onset of epileptic seizures,^130^ stroke^31,131,132^ and vascular dementia^32^ has exploited the putative property of ^55^Co as a PET tracer for calcium flux. The potential analogy to other aspects of calcium metabolism such as calcification have not been studied and the calcium-mimic hypothesis remains speculative. Notably, the ^55^Co biodistribution study performed by Mastren *et al.* lacks data on the pancreas, although prior studies in rats have shown some pancreas uptake.^133^ Unlike other radioactive trace metals which are mostly reabsorbed or retained by the kidneys, cobalt is readily excreted into the urine.

## Group 10 (Ni, Pt)

Group 10 transition metals have no known essential role in humans, therefore the main interest in studying their biology is in relation to their toxicology, as environmental contaminants or as metallodrugs. ^57^Ni (β^+^, 43.4%, *t*_1/2_ = 35.6 h) has been briefly explored as a PET imaging radiolabel for metal chelating drugs (doxorubicin).^134^ To our knowledge, only one study has actually used ^57^Ni to study the toxicology of nickel itself with intestinal absorption after oral administration estimated between 1.7 and 10% and uptake predominantly in the kidney and liver.^135^

^195m^Pt (γ, *t*_1/2_ = 4 days) is of particular potential interest to explore the pharmacology and pharmacokinetics of platinum anti-cancer drugs such as cisplatin. It is produced by thermal neutron irradiation of an enriched ^194^Pt target. Its emissions include several gamma photons (60–100 keV) that can be imaged by SPECT. Clinical trials with radiolabelled cisplatin are currently underway. Abundant Auger emissions also make ^195m^Pt, and ^193m^Pt (*t*_1/2_ = 4.33 days), potential therapeutic options.^136–138^

## Group 11 (Cu, Ag, Au)

Copper is an essential trace metal central to many biochemical and physiological processes, and disruption of its trafficking, accumulation and excretion is both cause and consequence of many pathologies, including inherited diseases such as Wilson's disease (WD), diseases of ageing such as dementias, and cancer. It is to be expected, therefore, that molecular imaging of copper trafficking using radiocopper might offer both important insights into the mechanisms underlying these key processes, and methods for diagnosis and monitoring of treatment. Indeed, radioisotopes of copper remain some of the most utilised radiometals described in this review. However, their applications to date have largely involved copper radionuclides as radiolabels for protein- and peptide-conjugates for PET imaging of specific molecular targets. Copper radionuclides with applications in nuclear medicine include: ^60^Cu (β^+^, *t*_1/2_ = 23.7 min), ^61^Cu (β^+^, *t*_1/2_ = 3.3 h), ^62^Cu (β^+^, *t*_1/2_ = 9.76 min), ^64^Cu (β^+^, 17.8%, β^−^, 38.4%, *t*_1/2_ = 12.7 h), and ^67^Cu (β^−^, 100%, *t*_1/2_ = 62.0 h). All except ^67^Cu offer a range of attributes useful in PET, while ^67^Cu has great potential for radionuclide therapy. Incorporation of these radionuclides into several copper(ii) chelators has been described,^139^ including a number of bifunctional derivatives which have permitted labelling of biomolecules such as antibodies and peptides. Redox and ligand exchange properties of copper itself are critical in some applications, notably in the bisthiosemicarbazone family of complexes, whose redox chemistry is key to their utility in PET imaging of hypoxia and perfusion.^140^ Both cyclotron-produced ^64^Cu and generator-produced ^62^Cu were utilised for the synthesis of radiocopper bis(thiosemicarbazone) complexes,^141–146^ but the short half-life of ^62^Cu is particularly exciting, presenting opportunities for repeated imaging studies with multiple tracers in the same patient. The ability to ship the ^62^Zn/^62^Cu generator from cyclotron sites to hospitals (^62^Zn, *t*_1/2_ = 9.3 h) allowed for the clinical use of ^62^Cu tracers.^143,145,146^ However, the most widely available and used copper radioisotope is ^64^Cu, which due to its versatile half-life (*t*_1/2_ = 12.7 h), established cyclotron production method and favourable positron energy (0.655 MeV) found multiple applications in clinical oncologic PET imaging with small molecules, peptides and antibodies.^147–152^

Due to the presence of β^+^/β^−^ emissions in the ^64^Cu and ^67^Cu decay schemes, and Auger electrons in the former, these radioisotopes lend themselves to radionuclide therapy. Therapeutic doses of ^64^Cu- and ^67^Cu-labelled tumour-targeting molecules^153–158^ or radiocopper salts^159,160^ demonstrated therapeutic efficacy in several preclinical tumour models, and some showed favourable tumour retention^161^ or partial therapeutic response^162,163^ in cancer patients.

These applications illustrate the breadth of interest in and utility of copper radionuclides as radiolabels for molecular imaging. However, their application to the study of the trafficking of copper itself have received much less attention and their potential is only recently being realised. Examples of such applications are discussed in the following sections.

### Visualising whole-body copper fluxes with ^64^Cu PET

**Fig. 6:**
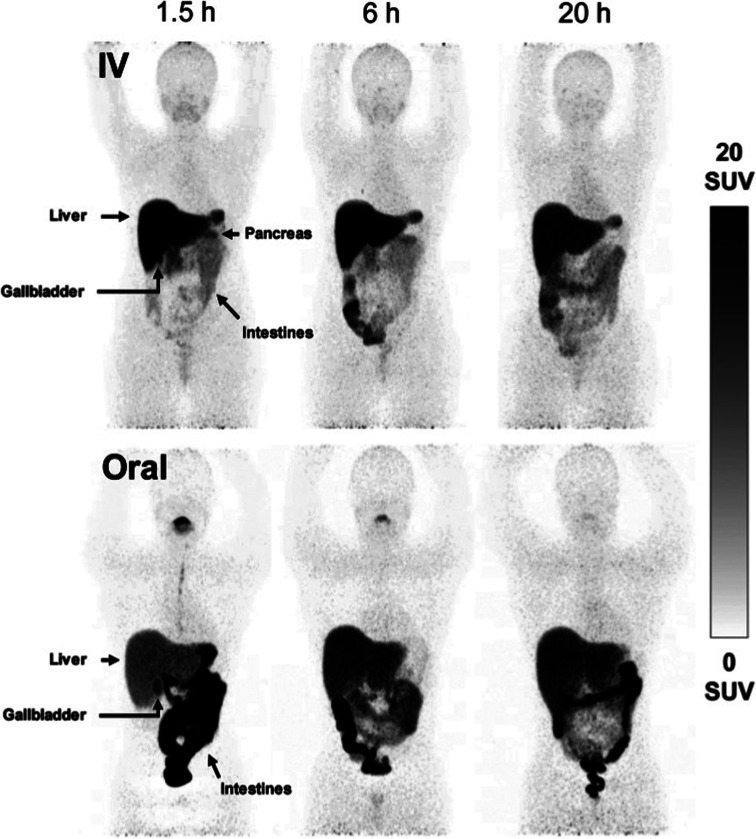
Comparison of i.v. and orally administered acetate-buffered [^64^Cu]CuCl_2_ in healthy human subjects. Whole-body MIPs in two healthy individuals, showing redistribution of ^64^Cu from the bloodstream to the peripheral organs with pronounced hepatic accumulation following i.v. injection of acetate-buffered ^64^Cu (upper panels), while the oral administration of ^64^Cu resulted in significant ^64^Cu signal in the intestines and slower distribution of ^64^Cu throughout the body due to limited absorption from the gastrointestinal tract. Adapted with permission under a Creative Commons Attribution (CC-BY) License from ref. 169, *EJNMMI Radiopharmacy and Chemistry*, copyright© 2020.

While PET imaging of copper trafficking is a relatively new field, radioactive copper isotopes have been used for decades to determine copper distribution after oral or i.v. administration in animals, followed by tissue and blood sampling and *ex vivo* gamma counting.^165^ Radiocopper blood clearance and excretion studies were also performed in humans to support the diagnosis of patients with Wilson's disease,^166–169^ an inherited disorder of copper metabolism. PET imaging with ^64^Cu-based tracers has been introduced to extend observation from specified tissues and body fluids to whole-body copper trafficking. The majority of preclinical and clinical PET studies have utilised oral or i.v. ^64^Cu formulations of copper chloride^164^ (the chemical form of ^64^Cu produced during the purification process). In some studies it has been buffered with acetate^170^ or citrate,^171^ or injected as [^64^Cu]Cu–histidine complexes.^172^ Upon i.v. administration, weakly chelated ^64^Cu associates with copper-transporting serum proteins – mainly albumin, which distribute copper to target tissues.^173,174^ However, how these different administration forms affect the kinetics of copper exchange between serum components and subsequent delivery to cells remains largely unknown. After i.v. administration, ionic radiocopper salts exhibit high liver uptake with hepatobiliary clearance, some spleen and kidney uptake, low brain uptake (at least at ^64^Cu imaging time-scales of several days) and barely detectable urinary excretion. A recent side-by-side comparison of i.v. and orally administered acetate-buffered ^64^Cu in healthy human subjects (Fig. 6)^170^ showed that oral administration led to lower activity in liver and peripheral organs, and higher in the intestines, presumably due to incomplete intestinal absorption.

### Cancer

**Fig. 7:**
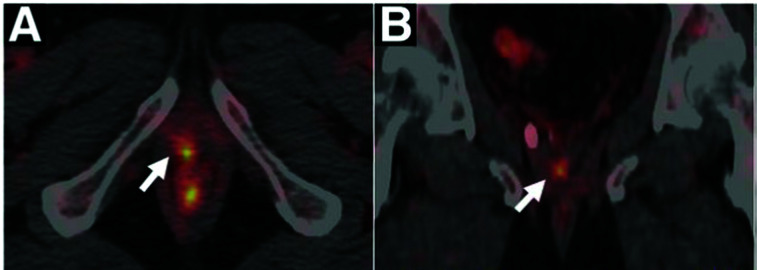
Clinical PET imaging with i.v. injected [^64^Cu]Cu–chloride to detect biochemical relapse in prostate cancer patients. The tumour is located in the pelvic region and is indicated with an arrow. This research was originally published in *J. Nucl. Med.*, Piccardo *et al.*^64^CuCl_2_ PET/CT in Prostate Cancer Relapse. *J. Nucl. Med.*, 2018, 59, 444–451. © SNMMI.^164^

Copper is required for cellular processes implicated in tumour growth, angiogenesis and metastasis.^175^ Due to their avidity for copper, some tumours can be visualised by PET imaging with i.v. injected ionic radiocopper. This has been demonstrated in preclinical models of hepatoma,^176^ hepatocellular carcinoma,^177^ melanoma,^159,178^ glioblastoma,^160,179^ neuroblastoma,^180^ prostate,^181,182^ head and neck^179^ and breast^183^ cancers. The utility of this approach for cancer diagnosis or staging largely depends on its anatomical localisation as [^64^Cu]Cu–chloride has high uptake in abdominal organs but low bladder accumulation. The low urinary excretion of [^64^Cu]Cu–chloride allowed for its use in the detection of primary tumour lesions in prostate cancer patients (Fig. 7),^164,184^ while its low normal brain uptake lends itself to visualising brain cancer lesions, demonstrated in patients with glioblastoma multiforme.^185^ In the latter case, it is unknown whether high radiocopper accumulation was a result of upregulated copper transport pathways in the tumour lesions or merely of disruption of the BBB, which is often reported in gliomas.^186^

The molecular determinants of high radiocopper accumulation in tumours are not fully understood. ^64^Cu uptake is partially dependent on the expression of high affinity copper uptake protein 1 (CTR1), which is the main known cellular copper importer. In a prostate cancer xenograft model, silencing CTR1 decreased tumour uptake of [^64^Cu]Cu–chloride by almost 50%,^182^ while CTR1 overexpression in breast cancer xenografts doubled [^64^Cu]Cu–chloride tumour uptake.^183^ However, the analysis of five cancer lines of various origin found no correlation between their CTR1 mRNA levels and *in vivo* uptake of [^64^Cu]Cu–chloride in xenografts.^179^ Apart from CTR1, radiocopper accumulation in tumours could be modulated by the expression of copper export proteins or intracellular copper scavengers. Since radiocopper in blood is largely associated with serum proteins, such as albumin, its delivery and retention in tumours could also be attributed to the local changes in the vasculature and lymphatic drainage affecting the accumulation of larger molecules.^187^ Increased ^64^Cu retention was also reported in the sites of traumatic brain injury^188^ or muscular injury^189^ but similarly to the studies in cancer models, the mechanisms of accumulation in the injured tissues were not elucidated.

Several groups have also appreciated the potential of ^64^Cu PET for visualising the changes in ^64^Cu trafficking following therapeutic interventions, which could be used to learn about their mechanisms of action, assess their efficacy and visualise side effects. For example, Parmar *et al.*^180^ used PET imaging with [^64^Cu]Cu–chloride to show that the experimental drug dextran-catechin targeting copper metabolism in neuroblastoma cells effectively reduced ^64^Cu uptake specifically in the tumour, without affecting ^64^Cu accumulation in other organs. Numerous other molecules, which either bind copper and remove it from the body (copper chelators) or exert toxicity in synergy with elevated intracellular copper, have been evaluated in preclinical cancer models with promising results.^175^ Some have been evaluated as adjuvant anti-cancer therapies in clinical trials.^190–192^ One such study incorporated ^64^Cu PET imaging to assess the tumour avidity for copper.^193^

### Wilson's disease

**Fig. 8:**
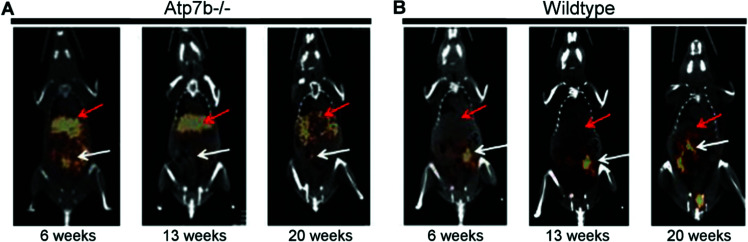
Preclinical ^64^Cu-PET imaging in a mouse model of WD. (A) Representative PET/CT images 24 hours after oral administration of [^64^Cu]Cu–chloride visualise hepatic copper overload and urinary excretion during disease progression in the Atp7B^−/−^ mouse model of WD, compared to reduced uptake in the wildtype control (B). Red arrows and white arrows identify ^64^Cu present in the liver and gastrointestinal tract respectively. Adapted with permission under a Creative Commons Attribution (CC-BY) License from ref. 199, *PLoS One*, copyright© 2012.

Wilson's disease (WD) is a recessive genetic disorder caused by mutation of the copper export protein ATP7B.^194^ It causes systemic copper overload, particularly in the liver and, most intractably, brain. The disease is often undetected due to limitations of current diagnostic methods^195^ and it is fatal if untreated.^196,197^ To diagnose WD, clinicians use a combination of blood and urine tests that have high false positive/negative rates.^197^ Confirmation of high copper content in the liver following biopsy is the most reliable biochemical test but it is invasive and prone to sampling errors, while genetic testing is challenging due to the presence of any of over 700 different ATP7B mutations.^194^ This motivated several groups to explore the possibility of using non-invasive PET imaging with ^64^Cu for WD diagnosis. Studies using ATP7B^−/−^ knock-out mice revealed that 24 hours after i.v.^198^ or oral^199^ administration of [^64^Cu]Cu–chloride, radiocopper liver overload and impaired biliary excretion was clearly distinguishable in this WD mouse model, compared to control mice. PET imaging with ^64^Cu was also used in longitudinal studies tracing whole-body copper fluxes during disease progression, demonstrating age-dependent changes in radiocopper liver uptake and urinary excretion^200^ (Fig. 8) or brain accumulation^201^ in the mouse model of WD. In the latter case however, brain uptake of [^64^Cu]Cu–chloride was very low, which made meaningful comparisons difficult. Clinical studies confirmed the utility of PET imaging as a tool for WD diagnosis. Compared to the healthy controls, WD patients had demonstrably impaired biliary excretion of ^64^Cu from the liver and as a result, significantly higher ratio of hepatic ^64^Cu retained at 20 hours and 1.5 hours after i.v. injection of [^64^Cu]Cu–chloride.^202^ It was possible to derive an uptake ratio value, which discriminated between the two groups without any overlap, thus presenting a tool to diagnose WD based on the measurable impaired hepatic copper clearance.

**Fig. 9:**
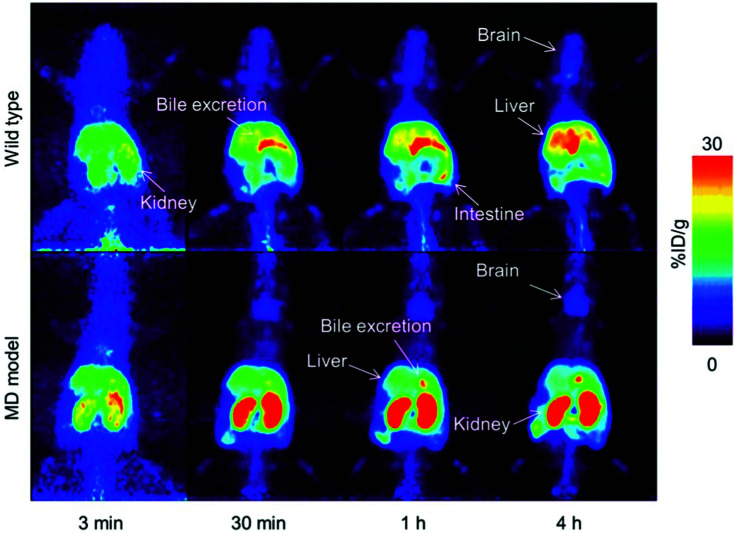
Imaging treatment response in a mouse model of Menkes disease with ^64^Cu-PET. Preclinical PET imaging with i.v. injected [^64^Cu]Cu–chloride to assess ^64^Cu redistribution after treatment with disulfiram and d-penicillamine. This research was originally published in *J. Nucl. Med.* Nomura *et al.* PET imaging analysis with ^64^Cu in disulfiram treatment for aberrant copper biodistribution in Menkes disease mouse model. *J. Nucl. Med.*, 2014, 55, 845–851. © SNMMI.^203^

Apart from the potential of ^64^Cu-based tracers for supporting WD diagnosis, they could also be used to monitor treatment response. PET imaging with [^64^Cu]Cu–histidine was used to demonstrate how injection of healthy hepatocytes reconstituted normal biliary excretion of ^64^Cu in a rat model of Wilson's disease.^172^ Nomura *et al.*^203^ used a similar approach in a mouse model of Menkes disease (another genetic disease of aberrant copper trafficking^204^) and with [^64^Cu]Cu–chloride PET imaging compared side-by-side how two different copper-modulating molecules, disulfuram and d-penicillamine, re-distributed ^64^Cu differently between organs (Fig. 9). These examples set a precedent for the integration of ^64^Cu PET in the preclinical drug development process, as well as in clinical trials for non-invasive assessment of experimental drugs or treatment regimens in individual patients. Copper chelators have been used for treatment of WD for decades^205^ and other experimental therapies are under development.^194^ Use of the short-lived radionuclide ^62^Cu may prove particularly useful in some of these potential applications, since it would allow for repeated scanning of the same subject, *e.g.* before and after therapy. With the advent of total-body PET and the improvements that this innovative technology provides,^2,206^ we anticipate studies taking full advantage of shorter-lived radionuclides.

### Neurodegeneration

**Fig. 10:**
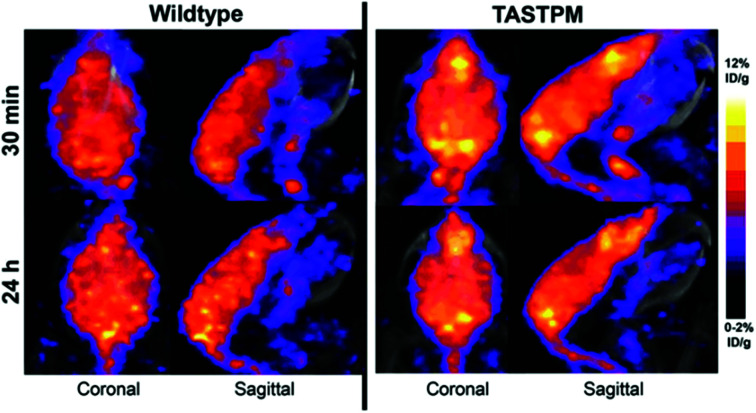
Imaging copper trafficking in Alzheimer's disease with ^64^Cu-PET. Preclinical PET imaging of the head after i.v. injection of [^64^Cu]Cu–GTSM demonstrating alterations in the brain and spinal cord copper clearance in a mouse model of AD (TASTPM). This research was originally published in *J. Nucl. Med.* Torres *et al.* PET Imaging of Copper Trafficking in a Mouse Model of Alzheimer's Disease. *J. Nucl. Med.*, 2016, 57, 109–114. © SNMMI.^218^

After the liver, the brain is the organ with the second largest copper content in the body,^207^ which reflects high requirement for copper for enzyme function,^208,209^ synaptic signalling^210^ and myelination of neurons.^211^ With its high rate of aerobic respiration, the brain is particularly vulnerable to redox stress so copper levels need to be very tightly controlled. This could be why brain exhibits remarkably slow kinetics of copper accumulation – upon i.v. injection of radiocopper in rats, the peak of brain accumulation did not occur until 13–17 days p.i., while other tissues were already in the radiotracer efflux phase.^212^ Due to the unique functions and kinetics of copper in this organ, it is of particular interest to non-invasively study brain copper trafficking *in vivo*, especially because copper imbalance accompanies numerous neurodegenerative diseases.^213,214^ Such studies could enhance our understanding of whether deregulated metal homeostasis is an underlying cause or consequence in particular types of neurodegeneration and whether changes are global or regiospecific, or acute or chronic. As shown in WD mice^201^ and in healthy mice,^215^ short term (<2 days) imaging with ionic ^64^Cu gives very low signal in the brain with uptake restricted to the ventricles. This inspired the use of an alternative approach – using a lipophilic tracer [^64^Cu]Cu–GTSM, a radiocopper bis(thiosemicarbazone) complex which, upon i.v. administration, non-specifically delivers ^64^Cu to tissues (including brain) and releases it intracellularly due to the shift in the redox environment. Other bis(thiosemicarbazone complexes such as [^64^Cu]Cu–ATSM and [^64^Cu]Cu–PTSM only dissociate under more hypoxic conditions,^216^ and therefore would not be expected to release ^64^Cu indiscriminately in perfused tissues, as does [^64^Cu]Cu-GTSM. This property of [^64^Cu]Cu–GTSM allows for subsequent observation of copper retention and efflux processes, without limitation of initially restricted delivery. PET imaging with i.v. injected [^64^Cu]Cu–GTSM demonstrated increased ^64^Cu brain uptake in the βPP/PS1 mouse model of Alzheimer's disease (AD).^217^ Using the same approach, changes in the ^64^Cu clearance kinetics and brain biodistribution of ^64^Cu were reported in two neurodegeneration mouse models: the TASTPM model of AD^218^ (Fig. 10) and in mice modelling Niemann-Pick C disease,^219^ a genetic lysosomal storage disorder. The exact intracellular fate of ^64^Cu released from the [^64^Cu]Cu–GTSM complex *in vivo* and how it compares with the fate of ^64^Cu delivered to cells *via* CTR1 (which is presumed to follow highly chaperoned endogenous intracellular copper trafficking pathways) remains unknown.

Silver does not have a known biological function but it has long been used in medicine due to its antimicrobial properties.^220^ An example of a silver-based medicine used nowadays is silver sulfadiazine, which is applied in ointment to disinfect burn wounds. Silver formulations, in metallic and nanoparticle forms, have also been used to coat medical implants and prostheses. However, pharmacokinetics of silver in the body is not fully described, nor are the mechanism underlying potential systemic leaching of silver from the sites of application (as an ointment or from medical devices).^220^ Silver radioisotopes such as ^104^Ag (β^+^, *t*_1/2_ = 69 min), ^104m^Ag (β^+^, *t*_1/2_ = 34 min) and ^106m^Ag (β^+^, *t*_1/2_ = 8.3 d) may be useful for imaging the fate of silver-based compounds.

Similarly to silver, gold has a long history in traditional medicine but also forms a basis of modern approved drugs, such as gold salt auranofin approved to treat rheumatoid arthritis.^221^ Another application of gold is in the synthesis of gold nanoparticles, which received much interest in the field of nanomedicine due to their versatility and stability in biological media. There is also considerable interest in repurposing gold compounds for cancer therapy and as antimicrobial agents. However, despite these applications, there is still no consensus on what the key molecular mechanisms underlying the therapeutic action of gold salts and their toxicity profile may be; knowledge gaps also exist in our understanding of the fate of gold compounds in the body.^221^ Some of these questions could be addressed by SPECT imaging of gold compounds using gold radioisotopes; indeed such approach has been demonstrated in imaging the pharmacokinetics of gold nanoparticles using ^199^Au (β^−^, γ, *t*_1/2_ = 3.2 d).^222^^198^Au (β^−^, γ, *t*_1/2_ = 2.7 d) has some gamma emissions that can be imaged with SPECT.

## Group 12 (Zn, Cd, Hg)

At least 10% of the human genome encodes zinc proteins, amounting to over 3000 proteins. In keeping with its importance, the homeostasis of zinc is regulated by an array of proteins that bind, store and release zinc to meet cellular demand. The ZRT/IRT-like protein (ZIP) family (SLC39A), consisting of 14 members, is responsible for elevating cytosolic zinc levels. It does this by importing zinc into the cell from the extracellular fluid, or through the release of zinc from organelles such as the endoplasmic reticulum and mitochondria. Export of zinc from the cell is controlled by a smaller family of cation diffusion facilitator proteins known as the zinc transporters (ZnT). This export includes moving zinc from the cytosol to the extracellular space, but also the compartmentalisation of zinc into organelles. Disruption of the expression and activity of these transporters, combined with zinc deficiency and overload, have been associated with several diseases including cancer,^223–227^ Alzheimer's disease^228,229^ and diabetes.^230–232^ Despite these hints of the pathological importance of zinc biology, its role in maintaining health and the regulation and dysregulation of their trafficking – from diet to tissues, tissues to tissues and excretion – in diseases such as dementias, cancer and diabetes – remain poorly understood.

Much effort has been invested in recent decades in the synthesis of fluorescence sensors that can be used *in vitro* to study zinc biology. An extensive library of such probes has been developed,^233–242^ combining Zn^2+^ chelating units, such as di-2-picolylamine (DPA), and a fluorescent reporter that, following zinc binding, initiates a change in emission properties (*e.g.* quantum yield and/or wavelength). Due to the limited tissue penetration associated with fluorescence, these probes have rarely been implemented *in vivo*. Recent efforts have been made to design medical imaging contrast agents to study zinc *in vivo*,^241,242,245–247^ and these have proved useful to study labile zinc pools. However, tools providing complementary information on the dynamic flux of zinc within the body are lacking. ^65^Zn (β^+^, *t*_1/2_ = 243.8 days) is the longest lived positron-emitting radioisotope of zinc and has the longest history as a research tool; it allows zinc biodistribution to be followed for over a year, which has seen historic use in the study of zinc absorption and metabolism in rodents^248,249^ and humans.^250,251^ However, its long half-life means ^65^Zn is unsuited to imaging and its use depends on tissue biopsy and blood/urine sampling. Its application in animals has diminished in recent years as its long half-life introduces problems associated with contamination and disposal. Nevertheless, ^65^Zn still remains an important radioisotope for studies zinc in plant biology,^252^ cell uptake/efflux and as a standard for the calibration of gamma-ray detectors.

**Fig. 11:**
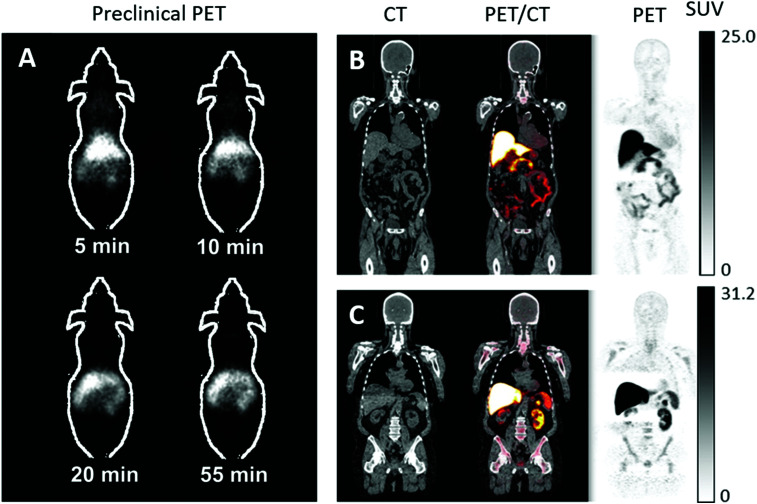
^63^Zn-PET can be used to study zinc trafficking *in vivo* up to 2 hours. (A) Serial small-animal PET images of healthy male B6.SJL mice after i.v. administration of [^63^Zn]Zn–citrate demonstrate predominant abdominal uptake. (B) CT, PET and fused PET/CT images are shown in a representative patient with Alzheimer's disease and healthy elderly participant (C) at 45 to 70 minutes p.i. of [^63^Zn]Zn–citrate. Uptake was observed in the liver, pancreas, spleen, kidneys, intestines and bone marrow with no qualitative differences between the groups. Adapted with permission from Degrado *et al.*^243,244^

In the last ten years, the production and translation of radioactive zinc isotopes more suitable for PET imaging have begun to emerge. The favourable positron emission of ^63^Zn (β^+^, 93%, *t*_1/2_ = 38.5 min) make it a desirable option for imaging zinc biodistribution *in vivo*. Degrado *et al.* successfully established a reliable production method for ^63^Zn using a low-energy cyclotron *via* the ^63^Cu(p,n)^63^Zn reaction using 14 MeV protons and an isotopically enriched ^63^Cu-copper nitrate liquid target. Cation exchange purification yielded ^63^Zn as [^63^Zn]Zn–citrate ready for *in vivo* administration. Preliminary PET imaging studies of [^63^Zn]Zn–citrate in mice following i.v. administration showed that, like other transition radiometals, ^63^Zn is rapidly cleared from the blood and is distributed primarily to abdominal organs (Fig. 11A). *Ex vivo* biodistribution at 60 minutes p.i. showed high uptake in the pancreas (standard uptake value (SUV) 8.8 ± 3.2), liver (6.0 ± 1.9), intestine (4.7 ± 2.1), and kidney (4.2 ± 1.3). This distribution was similar to that observed in humans with PET imaging (Fig. 11B and C). These initial human investigations identify potential future uses for this radionuclide, including imaging hepatic, renal and pancreatic function in the context of liver cirrhosis and diabetes respectively. Zinc dyshomeostasis has also been implicated in Alzheimer's disease, with a putative role in the aggregation of β-amyloid proteins that accumulate in brains of patients.^229^ Against this background, ^63^Zn-PET has been used in a small clinical study in patients with Alzheimer's disease (Fig. 11B). Although the penetration of ^63^Zn across the BBB is modest (brain SUV <0.5), uptake was enough to show that there was no significant difference in uptake between patients with Alzheimer's disease compared to healthy elderly volunteers. However, efflux of ^63^Zn from the brain was slower in the Alzheimer's cohort – in contrast to ^64^Cu where efflux was shown to be increased in a mouse model of Alzheimer's disease.^218,254^ This suggests that a tracer that facilitates increased brain uptake, similar in principle to the aforementioned studies with ^64^Cu–GTSM, using a lipophilic metastable zinc chelator (ionophore), might allow zinc efflux from brain to be imaged as a diagnostic pathological marker.

**Fig. 12:**
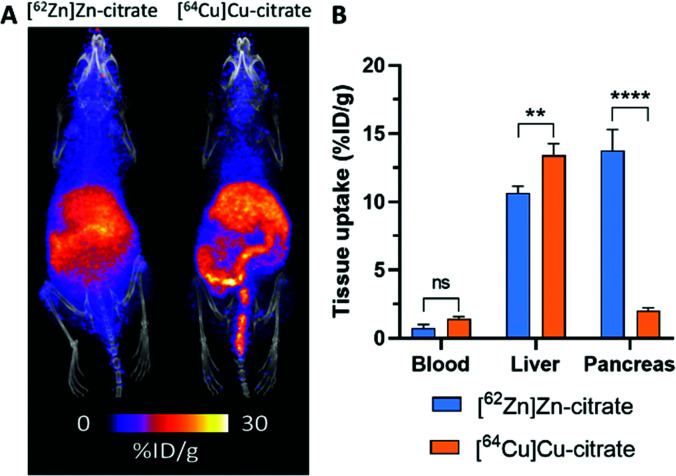
^62^Zn-PET can be used to study zinc trafficking *in vivo* over 2 days despite complex decay *via*^62^Cu. (A) Frontal maximum intensity projection (MIP) PET/CT images of female BALB/c mice (10–11 weeks old) injected i.v. with [^62^Zn]Zn–citrate (left) and [^64^Cu]Cu–citrate (right). (B) *Ex vivo* biodistribution at 24 h p.i. shows significant pancreatic uptake for ^62^Zn contrasting the lower uptake observed with ^64^Cu. Adapted with permission from ref. 253.

The short half-life of ^63^Zn limits applications to biological processes with a turnover time less than two hours. This is sufficient to monitor delivery of zinc from blood to tissues, but a longer-lived positron emitter is required to study excretion and redistribution of zinc from tissues to tissues over several days. ^62^Zn was discussed briefly in the previous section as the parent isotope in the ^62^Zn/^62^Cu generator. However, ^62^Zn also has applications of its own, and has previously been used to image pancreatic exocrine function^255^ and to label a few zinc-radiopharmaceuticals.^256,257^ Despite its useful half-life (9.3 hours), which allows for imaging over a few days, its complex decay *via*^62^Cu has therefore resulted in this radionuclide being overlooked as a viable option for studying zinc trafficking. In a sample of ^62^Zn, once equilibrium is reached, more than 95% of the emitted positrons are from the daughter radionuclide ^62^Cu, raising the question of whether *in vivo* imaging reflects the distribution of ^62^Zn or redistribution following decay to ^62^Cu. Recent observations by Firth *et al.* are beginning to address this question.^253^ The authors surmised that after 1 h p.i., each positron emitting radionuclide has spent >98% of its *in vivo* lifetime as ^62^Zn (a consequence of the 60-fold longer half-life of ^62^Zn compared to ^62^Cu), and unless ^62^Cu is extremely rapidly redistributed following conversion, then the vast majority of positrons should reflect trafficking of zinc, not copper. Preclinical PET investigations with [^62^Zn]Zn–citrate in mice (shown in Fig. 12A) demonstrated a similar biodistribution to [^63^Zn]Zn–citrate at 1 h p.i. but not to [^64^Cu]Cu–citrate; notably, uptake of ^62^Zn in the pancreas was much greater than the ^64^Cu control. Greater accumulation of ^62^Zn than ^64^Cu was also noted in the heart, spleen, bone, brain, salivary glands and prostate and seminal vesicles. These differences are consistent with the hypothesis that ^62^Zn is handled *in vivo* primarily as zinc and not as copper. The half-life of ^62^Zn (*t*_1/2_ = 9.3 hours) offers longitudinal imaging beyond the 2 hour imaging window of ^63^Zn, up to 2 days. ^62^Zn activity at 24 h p.i. was decreased in liver, pancreas and kidney, but was increased in the brain. This radionuclide therefore provides an important tool for imaging zinc trafficking *in vivo* and can be distributed nationally owing to its longer half-life. Monitoring the kinetics of zinc at very early time points remains complicated thanks to the complex decay of ^62^Zn, therefore a partnership between ^63^Zn, for investigations at early time points, and ^62^Zn, for imaging longer periods of time, may help address important biological questions.

Implementing radiozinc to image zinc homeostasis in cancer is also of significant interest. The large body of evidence that supports the decline in endogenous zinc with prostate cancer suggests that zinc may be a useful diagnostic biomarker.^223–226^ However, diagnostic PET is typically a hot-spot technique, and given that radiozinc delivery is likely to be reduced in prostate cancer, a cold spot representing cancerous tissue compared to high background may limit its clinical utility. Preclinical studies to investigate this are warranted, as well as in other cancers where zinc homeostasis is dysregulated such as breast and pancreatic cancers.

Group 12 also contains two metals that are not biologically essential but are known toxic environmental pollutants: cadmium and mercury. Cadmium toxicity can manifest in adverse effects ranging from renal failure, bone demineralisation and cancer.^258^ It is a pollutant of particular concern due to its ubiquitous presence in the industrial environment and its long half-life in human tissues (10–30 years in the kidney). Nuclear reactor production of ^115^Cd (β^−^, *t*_1/2_ = 2.2 days) was described in the context of manufacturing ^115m^In generators, since ^115m^In is its decay product.^259^^115^Cd use was also reported in *in vitro* transfer studies in human blood cells.^260^ Another radiocadmium isotope, ^115m^Cd (β^−^, *t*_1/2_ = 44.6 days), was used as a label for cadmium metabolism studies in humans.^261^ Both radionuclides are unsuitable for imaging.

Mercury is toxic upon inhalation, ingestion and dermal exposure. Its organic form methylmercury is a potent neurotoxin.^262^ Mercury has been listed by the World Health Organization (WHO) as one of the ten chemicals of major public health concern (along with three other metals/metalloids: arsenic, cadmium and lead), which together contribute to a significant disease and death burden worldwide.^263^ Gamma emitting ^197^Hg (γ, 77 keV, *t*_1/2_ = 64 h) and ^203^Hg (β^−^, γ, 279 keV, *t*_1/2_ = 47 days) were historically used in preparation of radiotracers for brain tumour and kidney imaging,^264,265^ or for radiotracer distribution studies.^266,267^ Its potential as an imaging tracer or label could be harnessed to study the toxicokinetics of mercury and its compounds and potential chelation therapies.

## Groups 13–18 (Pb, As, F, Cl, I)

The elements of groups 13–18 comprise both metals not known to be essential in humans, and non-metals that include some essential elements; hence they are somewhat tangential to this review. Nevertheless several of them represent important links between radionuclide imaging and essential element biology and hence warrant brief discussion here. Radioisotopes of carbon (^11^C),^268^ nitrogen (^13^N),^269,270^ fluorine (^18^F)^271^ have all seen varied use in nuclear medicine for radiolabelling small molecules, although the range of tracers labelled with ^13^N and ^15^O is very narrow because of their short half-lives (10 minutes and 2 minutes, respectively). ^11^C (β^+^, 100%, *t*_1/2_ = 20 min) allows for the synthesis of a very wide range of radiolabelled analogues of biomolecules including key nutrients such as glucose, amino acids, fatty acids and vitamins for *in vivo* PET imaging.

The classification of fluorine as an essential element is contentious; certainly it has a role in stabilising the mineral component of teeth. When injected in the form of fluoride ions, ^18^F accumulates quickly at sites of active bone mineral remodelling, such as bone cancers and metastases;^272^ thus, in principle, [^18^F]fluoride offers a means to use PET imaging to study fluoride trafficking in other pathological contexts such as calcification in arteries and atherosclerotic plaque (which has recently become a clinical reality),^273^ or specific trafficking mechanisms (if they exist) in healthy subjects. This application has not yet been implemented. Generally, ^18^F is used as a radiolabel for organic molecules. In the context of PET imaging of metal trafficking, a ^18^F radiolabelled fluorescent zinc sensor, consisting of a small lipophilic chelator that can enter cells passively and becomes trapped within them when bound to zinc ions, has recently been reported with the goal of non-invasively imaging endogenous Zn pools *in vivo* in diseases such as prostate cancer.^242^ The biological validation and potential of such a radiolabelled probe is yet to be reported. Given the plethora of fluorescence probes for the detection of metals, other ^18^F radiolabelled metal sensors can be envisaged.

Iodine is an essential trace element, forming a constituent of thyroid hormones. Imaging the trafficking of iodine using gamma-emitting radionuclides ^123^I (γ, 159 keV, *t*_1/2_ = 13 h), ^125^I (γ, 35 keV, *t*_1/2_ = 60 days), and more recently positron-emitting ^124^I (β^+^, 23%, *t*_1/2_ = 4.2 days), usually in the form of iodide, is one of the oldest manifestations of nuclear medicine, both diagnostically and therapeutically (using the beta-emitter ^131^I, *t*_1/2_ = 8.1 days) in thyroid disease and thyroid cancer. Its accumulation in thyroid, stomach and salivary glands has been attributed since the 1990s to activity of the sodium–iodide symporter in these tissues.^274^ Hence, radionuclide imaging with iodine radionuclides offers utility in basic studies of iodine trafficking mechanisms, which has not been widely exploited outside clinical diagnosis and therapy.

Chloride is the major extracellular anion in the human body and is responsible for osmotic pressure, acid–base balance and gastric HCl production. The short half-life of ^34m^Cl (β^+^, 53%, *t*_1/2_ = 32 min) necessitates on-site production, and reliable production methods to facilitate this are currently under development. ^34m^Cl provides an underutilised synthon that will allow the synthesis of many unstudied chlorine-containing biological compounds. ^34m^Cl is also of interest, though yet to be studied, to image chlorine transport *in vivo*. Cystic fibrosis, seizures, osteopetrosis and myotonia are associated with disruption to chloride homeostasis, and our understanding of the underlying biology of these conditions may benefit from PET imaging studies with ^34m^Cl.

Other elements in groups 13–18 deserve mention which, despite having no essential biological role, are of interest in the metallomics field because of their toxicity and industry- or environment-related disease. Lead is one of the key metal environmental pollutants, giving rise to renal, cardiovascular and brain toxicity, and children are the most vulnerable population to lead neurotoxicity.^262^ Lead has radionuclides with promise as a theranostic pair – ^212^Pb (β^−^, *t*_1/2_ = 10.6 h) is of interest as an *in vivo* generator of the alpha emitter bismuth-212 (*t*_1/2_ = 60.6 min) and is in early stages of development for radionuclide therapy of cancer when conjugated to tumour-targeting biomolecules,^275^ and ^203^Pb (γ, 81%, 279 keV, *t*_1/2_ = 51.9 h) is a gamma emitter being developed as an imaging counterpart. Availability of ^203^Pb may stimulate imaging studies of the trafficking and biodistribution of lead assimilated from the environment. Similarly, the ready availability of ^201^Tl, discussed in earlier sections as a potassium analogue, could be used to study the toxicology of thallium.

Gallium radionuclides have already been discussed previously in the context of imaging iron trafficking.

Arsenic is of interest both as an environmental toxin, exposure to which causes skin lesions, cardiovascular effects and several types of cancer,^276^ and as the anticancer drug arsenic trioxide.^277^ Arsenic radionuclides have received some attention for nuclear medicine applications (*e.g.* as a radiolabel for cancer-targeting molecules or nanoparticles),^222,278,279^ but have seen relatively little use to study the pharmacology or toxicology of arsenic. Among the 29 known arsenic radioisotopes, six have a half-life suitable for use in studying arsenic biology; three of these are positron emitters: ^71^As (β^+^, 28%, *t*_1/2_ = 2.72 days), ^72^As (β^+^, 88%, *t*_1/2_ = 1.08 days) and ^74^As (β^+^, 60%, *t*_1/2_ = 17.77 days), and can therefore be imaged with PET. ^71^As and ^72^As can be produced by alpha bombardment of a gallium target or *via* proton bombardment of enriched germanium. Alternative production of ^72^As using a ^72^Se/^72^As generator has also been proposed with similar if not greater radionuclidic purity compared to germanium bombardment,^280,281^ but the relatively short-lived parent (*t*_1/2_ = 8.4 days) limits its use to one month. The long half-life of ^71^As and ^72^As is comparable to the established PET radionuclide ^89^Zr, suggesting similar applications such as attachment to antibodies and proteins for receptor mapping, with the advantage over ^89^Zr of fewer high-energy gamma emissions. However, arsenic radiochemistry is far less developed and worldwide production and supply of clinical grade ^72^As is not yet established. Growing availability of radioarsenic suitable for radionuclide imaging will facilitate the study of arsenic biology, particularly to better understand the mechanisms underpinning arsenic poisoning and the mechanism of action and trafficking of the anti-cancer drug, arsenic trioxide.

## Conclusion and future directions

Interest in the biological roles of trace metals (and other trace elements) has resurged in recent years alongside developments in analytical methods, both to improve understanding of the relevant biology and to diagnose and treat disease. Methodological developments such as ICP-MS which have been adapted to 2D imaging (*e.g.* LA-ICP-MS, SIMS *etc.*) to map elemental distribution at cellular level in cells and tissue slices have been particularly influential. Such methods can deliver a snapshot of *in vitro* or *ex vivo* distribution at a single time point on selected tissue samples. Recent developments in production and availability of radioisotopes of biological trace metals, or their surrogates, and toxic metals, are now making it possible to perform molecular imaging of trace metal trafficking at the whole body level in living subjects, including humans. Such radionuclides have conventionally been used largely as radiolabels for larger biomolecules, but by using them to study the biology of the metals themselves, PET and SPECT imaging with these radionuclides can now offer new and complementary insights with a window on the dynamic processes that lead to element distributions, non-invasively and longitudinally. This review has outlined examples of current and potential uses of such radionuclides. By collaborating with centres that host molecular imaging facilities, metallomics and bioinorganic chemistry researchers can now transition their studies from *in vitro* and cell level to *in vivo* and clinical investigations. Such studies have progressed to clinical translation in diseases involving changes in copper trafficking, using ^64^Cu, and the potential is now clear for similar progression to *in vivo* studies with radioisotopes of zinc, manganese and other essential or toxic elements and metallodrugs. We hope that metallomics researchers will be encouraged to consider making use of these molecular imaging tools to complement elemental analytical tools, to study *in vivo*, and ultimately in humans, the effects of genetic manipulation, disease and therapy on metal trafficking.

## Author contributions

G. F. and P. J. B. were responsible for the concept of this article. Sections on metals within individual groups were distributed among all the authors, G. F. orchestrated the individual sections into a coherent manuscript. G. F., J. E. B., J. J. B. and P. J. B. contributed to editing and reviewing the manuscript. All authors approved the final version of the manuscript.

## Conflicts of interest

There are no conflicts to declare.

## Supplementary Material
